# 

**Published:** 1912-04-06

**Authors:** 


### Buildings Contemplated and in Progress.

Caerphilly.—Tenders are invited for a complete electric-lighting installation at the Energlyn Isolation Hospital, Caerphilly.

Clacton-on-Sea.—Eight tenders have been received for a home and lodge at Clacton-on-Sea for physically defective children. The lowest amounts to £11,275; the highest is £13,185.

Conisborough.—The Doncaster and Mexborough Joint Hospital Board have agreed to extend the Isolation Hospital at an approximate cost of £5,000.

Glasgow.—Tenders are invited for the several trades in connection with the erection of another medical block at the Glasgow Royal Infirmary. Mr. Miller, A.R.S.A., is the architect.

Guisborough.—Plans have been approved by the Guardians for the erection of a nurses' home and a children's home; the plans are now submitted to the Local Government Board.

Haslingden.—The Guardians have appointed an architect (Mr. H. Ross) to submit details showing the cost of adapting certain wards at the old infirmary for sanatorium purposes on the lines suggested by Dr. Nathan Raw.

Hunstanton.—Messrs. MacAlister and Skipper, of Cambridge, are architects for the additions, etc., to the Hunstanton Convalescent Home. A tender of close on £3,000 has been accepted for the works.

London.—Additions, including the erection of a porter's lodge, dispensary, and an extension of nurses' home are proposed by the Lambeth Guardians for the Kennington Road Infirmary. Mr. F. A. Powell, F.R.I.B.A., is the architect, and tenders are now invited.

Lyminge (Kent).—Nineteen tenders have been received for the erection of a female infirmary and nurses' home at the Elham Union Workhouse. The lowest (provisionally accepted) amounts to £4,255, the highest to £7,447. The architect is Mr. A. R. Barles, of Folkestone.

Park Prewett.—Tenders are invited for foundation works at the new asylum near Basingstoke (Hants). It will be recalled that the architects are Messrs. Hine and Pegg, of London, and particulars for tendering may be had from them on deposit of £5 (returnable).

Stafford.—The Board of Guardians has resolved to apply to the Local Government Board for an order combining the several unions in North Staffordshire for the provision of separate accommodation for epileptics and feeble-minded.

Swansea.—The architect (Mr. Hind) has been instructed by the Swansea and Merthyr Joint Asylum Committee to prepare a design for asylum premises to accommodate 1,000 patients, a villa for sixty patients, and two semi-detached villas as sanatoria. It is probable that the scheme in its entirety will not be proceeded with at once.

Warrington.—The Guardians have given instructions for the preparation of plans for a new infirmary to accommodate 160 patients.

Willesden.—The Guardians have decided to erect a home to accommodate about thirty nurses.

Winsley.—The Management Board of Winsley Sanatorium have decided to spend £9,000 on an enlargement.

### OUR BUREAU OF INFORMATION.

### Six Months' Work of the Bureau.

The first beginning of any new department must of necessity be tentative. The response may not come at once, but at the end of six months there is evidence to show whether what we have to offer is what the public wants.

Judged by this practical test, we can honestly say that our Bureau of Information has justified its existence. We have been able to satisfy ourselves on several points. First, that there is a fund of kindness that cannot be tested by institutional tests. Appeals which might have seemed so hopeless as hardly to be worth printing have never failed to meet with a response. The experience of the Bureau makes it clear to us that if the appeal comes the response will come also. There are those who will give help for nothing, and many others who will give house-room if the sufferer can pay the cost of food.

We have proved also that among those who keep nursing-homes for profit there are many whose terms are by no means so prohibitive as is generally supposed. There are many who wish to meet fairly the needs of people of moderate income, and some who, as one of them has expressed it to us, "set the small fees of the poorer against the large fees of the richer."

We have proved also that, especially in the country, there is much information wanted which is not readily accessible to our correspondents—information as to training in various callings, salaries, and prospects, which is easily obtainable by those in touch with the occupations in question, but not to others. To some of these queries the answer was easy; in other cases special information had to be obtained; but we can say that no one who wanted information as to a particular kind of work or of his fitness for it, has been left unanswered. And the answers given are careful and authoritative.

We have, then, proved that the Bureau is a useful department of The Hospital. It is also one that is capable of immense extension. And the extension depends on our readers. What they ask we will answer; what they seek we will do our best to find for them; and with the experience of the last six months we have little doubt of being able to meet their requirements.

### Needs and Helpers.

Home for Girls with Hip-disease.

"F. M. D." writes: Can you tell me of any Home (with or without payment) where two girls of fifteen, who are suffering from chronic tuberculous hip-disease, could be received? In or near London preferred.

Swedish Exercises.

"Mitre" writes: Could you give me the names of any books describing the "crawling exercises" recommended by Professor Klapp?

[Funktionelle Behandlung der Skoliose, by Professor Klapp (price 5s. 6d.), can be procured at Siegle and Co., Ltd., foreign booksellers, 2 Langham Place. There has also, we believe, been an article on the subject in the *Journal of Physical Training*, edited by Mrs. Adair Impey, Cropthorne, King's Norton, Birmingham. The exercises are taught by Dr. Mary Coghill Hawkes at the Swedish Institute, 106 Cromwell Road, S.W.]

Home for a Mental Case.

"A. B. C." writes: Could you help me, through your Bureau, to get into a small Home one who is mentally deficient? Her father, who is in business, could pay £1 1s. a week. Patient is about forty-three, gentle, well-behaved, silent in company, and can do light work such as dusting and bed-making, but is forgetful.

Removal of Superfluous Hairs.

"Nemo" writes: Can you tell me where in Manchester or Liverpool I would be likely to get *x*-ray or other treatment for the permanent removal of superfluous hairs? I would prefer a hospital.

### OUR LIST OF APPROVED HOMES.

"Clovelly," Kedleston Road, Derby. Home for undeveloped children and mild mental cases. Principal, Miss Martin, fully certificated nurse. The home is at the extreme verge of the town, and overooks parks and fields. The children's nurseries are quite apart from the rooms occupied by adult patients.

Ivy House, Pidley, Huntingdon. Principal, Miss F. M. Moles. Children, sick, ailing, backward, and slight mental cases. Large garden, open-air school in summer. Situation, 199 feet above sea-level.

Ifield Lodge, Uckfield, Sussex. High situation with extensive views. Principal, daughter and sister of clergymen. Principal wishes to adopt child if £1 a week guaranteed, or would take Indian children.

73 Royal Parade, Eastbourne. Principal, Sister Collins. Personally known to member of The Hospital staff. All cases.

"The Haven," 6 Holly Park, Finchley, N. Principal, Miss Jessie Holmes, M.R.B.N.A.

"Mascot," Seaton, Devon. Principal, Nurse D. Mills. South aspect, quiet situation. All ordinary cases, also permanent, accouchement, and delicate children.

"Cotswold," Ilfracombe. Principal, Miss Catherine Layton. Small Home for invalids and chronic patients. Mild climate.

"Woodmancote," Woodland Vale Road, St. Leonards-on-Sea. (Tel. 611 Hastings.) Matron, Miss Reinberg. All ordinary cases, chronics, and convalescents. No infectious or phthisis cases received. Good operating theatre. Terms from 3 guineas a week.

13 St. Vincent's Road, Westcliff-on-Sea. Principal, Mrs. Steele. Senile and mild mental cases.

45 Clarence Square, Brighton. Principal, Sister Miriam. Surgical, medical, and chronic. Large sunny rooms. Acute cases, £5 5s. per week; chronic and convalescent, 2½ to 3 guineas per week. Children received.

"The Orchard," Haslemere. Principals, Sister Herbert and Miss Maurice. Convalescent home for ladies and children. Surgical and nerve cases needing special care received. House situated 600 feet above sea-level, in the neighbourhood of pinewoods. Terms: Ladies from three guineas a week; children from two guineas a week.

4 Magdalen Road, St. Leonards-on-Sea. Principal, Miss Moody. Medical, surgical, and convalescent cases. Good operating theatre. Permanent cases received. Terms from three guineas per week.

Invalids' Home, 3 Milton Road, West Hartlepool. Principal, Sister Arnold. Medical, surgical, and chronic cases. Operating theatre. Bright convenient house, in quiet road near sea. Fees moderate. Sister Arnold also visits patients in their own homes.

### Rules for Correspondents.

Everyone who uses or wishes to help our Bureau of Information must be kind enough to observe the following rules:—

(1) Postal replies cannot be given. Exception will only be made in very special cases at the Editor's discretion.

(2) Queries or answers relating to different cases must be written, or preferably typed, on separate sheets of paper, and the writing must never be crossed.

(3) Questions and replies received not later than Wednesday will, as far as possible, be dealt with in our issue of the following Saturday week.

(4) Letters must have attached the name and address of the sender, with a pseudonym for publication if preferred.

(5) Applications referring to non-dependents where the need relates to business or employment must be accompanied by a postal order for 5s., when the requirement shall have publicity in these columns.

(6) All applicants for insertion in our list of Approved Homes must send a registration fee of five shillings. The omission of this leads to delay and gives avoidable trouble.

(7) *All communications* for this department *must be accompanied by the coupon* to be found at the bottom of the third page of the cover on the inside, and should be addressed to the Editor of the Bureau of Information, c/o The Hospital, 29 Southampton Street, Strand, London, W.C.

Resident Medical Officer.

[We have great pleasure in publishing Dr. Melvin's letter, but a careful perusal of it and of our report will show that he has not dealt with most of the points raised, except that relating to the reconstruction of the central block, which he admits is necessary.—Ed. The Hospital.]

### Gifts and Bequests.

Sir Clifford Cory, M.P., has given £400 to the West Cornwall Infirmary.

Mr. Charles Buchel, of Brighton, has left £250 to the Sussex County Hospital.

Mr. Isaac Julius Weinberg has left £200 to the Dundee Convalescent Home, Barnhill.

The Rev. John Larking Latham has bequeathed £350 to the Victoria Hospital, Dover.

Miss Bell, of South Marston, has bequeathed £1,000 to the Victoria Hospital, Swindon.

Mr. Henry J. Legge, of Surbiton, has bequeathed £1,000 to the Surbiton Cottage Hospital.

A sum of £115 has been raised by the annual ball at the Warneford General Hospital, Leamington Spa.

Three hundred pounds has been left to the East Sussex Hospital, Hastings, by the late Mary Jane Day, of Bexhill.

Mrs. Ellen Dyke Frost has left £1,000 to the Leicester Infirmary general fund, and £1,000 for the Children's Hospital.

From the estate of the late Mr. Charles Turner a further £1,000 will be handed over to the Leicester Infirmary.

Mrs. Ellen Penn has bequeathed £3,000 to the Miller Hospital, Greenwich, and £2,000 to St. John's Hospital, Lewisham.

Miss Maria M. Paxton, of Bournemouth, has left £500 to the Royal Victoria Hospital, Bournemouth, and £500 to the Firs Home.

Mr. Wm. Palgrave Wood has left £10,000 each to the Liverpool Bluecoat Hospital and the Liverpool Seamen's Orphan Institute.

Surgeon-General Sir John Andrews Woolfryes has left £500 each to St. George's Hospital and to the Royal Home and Hospital for Incurables.

The Chelsea Hospital for Women has received £200 from Mr. R. Nivison, and a £10 note which was stealthily placed in the letter-box from an anonymous donor.

Sir James Puckering Gibson, Bt., M.P., has bequeathed £2,000 to the Edinburgh Royal Infirmary and £1,000 to the Royal Victoria Hospital for Consumption.

Mr. Alfred Bell, of Newcastle, has left £2,000 to the Royal Victoria Infirmary, Newcastle, £2,000 to the Hull Infirmary, and £1,000 to the Ingham Infirmary, South Shields.

Mr. George Hahlo, of Manchester, has bequeathed £5,000 to the Manchester Hospital for Consumption and Diseases of the Throat and Chest, to endow a ward to be named after him.

Mr. James Cook, of Weston-super-Mare, has left the residue of his estate, amounting to about £36,000, to be divided between the Taunton and Somerset Hospital, the Bridgwater Hospital, and the West of England Sanatorium at Weston.

Mr. Arthur James has promised a further £2,000 towards the Warwickshire memorial to King Edward. He had already headed the subscription list with £500. The committee can now proceed to build the sanatorium for consumptives.

Mr. Nivison has given a donation of £200 to the London Fever Hospital, N. Sir George Lewis has left £50 each to the following hospitals: Charing Cross, the German, Guy's, King's College, the London, the Middlesex, the Royal Free, the North London, the Victoria Hospital for Children, St. Mary's, and St. Thomas's.

### Appointments.

Dr. McFetridge has been appointed non-resident medical officer by the Coventry Board of Guardians.

Dr. Hugh R. Phillips, M.D., Ch.B., has been appointed to fill the vacancy caused by the resignation of Dr. J. D. E. Mortimer as anæsthetist at the West End Hospital for Diseases of the Nervous System, which the latter has held since 1896. Dr. Phillips is anæsthetist to the Italian Hospital, and was formerly hon. physician and hon. anæsthetist at the Chest Hospital, Margaret Street, and civil surgeon in the South African War. Mr. L. M. Roath, M.B., B.C., and Captain A. Crichton Lupton, M.B., C.M. (1st Life Guards), have been appointed clinical assistants to the out-patients' physicians, and Mr. E. B. Gunson, M.D., Ch.B., has been appointed house physician for six months.

### The Week's Novelties.

### HENNESSY'S BRANDY.

(James Hennessy, Cognac.)

We have lately been favoured with samples of the brands manufactured by the well-known firm of distillers, Messrs. James Hennessy and Company, a firm that dates from 1765, when Richard Hennessy, third son of the squire of Ballymacmoy, started the business in Cognac. The reputation of the firm is now so firmly established that it is unnecessary to do more than refer to the varieties of brandies which this house exports. There are seven varieties. The one star is a six-year-old grape brandy; the two star is three years older; the three star is 12 years old; the V.O. brand is 15 years old, and then in succession follow the 25, the 40, and the 70 year old brands. These are all the same brands; they differ only in age, which has mellowed them and improved their quality. The admirable 70 years old liqueur brandy is beyond the means of most of us; from a purely dietetic and medicinal point of view we doubt if it is much superior to the 25 year old brandy. This latter and the well-known three star brand are the most popular, perhaps, and it is with these that we are chiefly concerned now.

A brandy of the type represented by Hennessy's Three Star is a household remedy that ought to be in every family. As a medicine its value is immense, for it combines the stimulating properties of the purest form of alcohol with the equally valuable properties of the numerous essential oils. As a dietetic drink it is, we consider, far superior to whisky. It is an adjuvant to digestion. Recent dietetic experiments have proved that the ingestion of a moderate quantity of alcohol in combination with such essential oils and bye products as are contained in "pot still" mixtures materially promotes the digestion and assimilation of proteids and carbohydrates. We may, therefore, take it that, to those who can use and not abuse it, brandy is a valuable dietetic aid. It is also a very pleasant tasting form in which to take the stimulant. All the brands sent to us have a very marked bouquet, and although there is an appreciable difference between them, they are essentially the same in so far that they are absolutely pure brandies. The firm issues a very interesting booklet ("Brandy: The Heart of the Grape") which we cordially recommend to every medical man. In these days, when the teetotal agitation is abroad even in the profession, no apology is necessary for drawing attention to the solid value of the products of the grape, and this is done in an admirable manner in the booklet just referred to. Messrs. Hennessy's brandies have won a well-deserved reputation for excellence and quality, and these excellent characteristics are fully maintained by the samples which we have just tested, and which are in every way samples of what pure, honest grape brandy should be.

We hope in a later issue to deal with the value of Hennessy's V.O. brandy as a liqueur when we consider the dietetic value of liqueurs in general.

### "COLLOSOL" ARGENTUM AND "COLLOSOL" HYDRARGYRUM.

The method of employing as therapeutic agents metals in a colloid state has made considerable advances, and with the perfection now reached by these preparations it is certain that hospital practitioners at any rate will use these agents more and more. We have had the opportunity of examining two preparations supplied by Messrs. Oppenheimer and Sons, namely, the "Collosol" Argentum and the "Collosol" Hydrargyrum, and there is no doubt that they represent an important advance in reliability and therapeutic efficacy. These special colloid solutions of these metals are manufactured solely for Messrs. Oppenheimer in the Crookes laboratory. These solutions are very stable, and are not precipitated by the blood. As regards their efficacy, the clinical records available up to date show that they are superior in their results to other colloid solutions in the treatment of bacterial infections, while their toxic effects on the human system are practically nil. The importance of this last feature is so obvious that further comment is needless.

### "THE NEW MULTOSTAT."

A new pamphlet just published by the Sanitas Electrical Co., Ltd., manufacturers of *x*-ray and electro-medical apparatus (offices and demonstration rooms, 61 New Cavendish Street, London, W.) describes several improvements in the design and construction of the Multostat, a "Universal Machine" for the medical application of the various currents to the human body. Not only, the makers state, is there no possibility of earth-shocks, but by means of a special condenser fitted in the galvanic circuit a true galvanic current is obtained, whereas in other universal machines, without this condenser, strong fluctuations of the galvanic current appear, with tetanic effects, harmful, and sometimes dangerous. Other improvements are that there are no openings in the base-plate or motor-case, and therefore no possibility of dust collecting in the internal mechanism, and in the new precision type turning rheostats, which provide the most smooth and accurate regulation of the strength of the various currents. A copy of this booklet will be sent free to any medical man on request.

